# Supplementary material for: Socioeconomic, Demographic, and Environmental Factors May Inform Malaria Intervention Prioritization in Urban Nigeria
Source: Int J Environ Res Public Health. 2024 Jan 10;21(1):78. doi: 10.3390/ijerph21010078 (PMC10815685; doi:10.3390/ijerph21010078)
Supplement: Supplementary file 1 [file ijerph-21-00078-s001.zip › ijerph-2693753-supplementary.pdf]

Supporting Information

S1 File: Supplementary appendix for Chiziba et al. "Socioeconomic and environmental factors may inform malaria intervention prioritization in urban Nigeria."

Contents

Test positivity rate by urban cluster, month and year of survey 2
Socioeconomic factors 3
Descriptive analysis 3
Sampled clusters had low levels of post-primary education, fell within the rich wealth quintiles and had improved housing conditions 3
Correlation analysis for socioeconomic covariates 4
Bivariate analysis 5
For most indicators of socioeconomic status, the lowest values correlated with higher levels of malaria positivity and test positivity rates 5
Demographic variables 9
Descriptive analysis 9
Cluster populations were generally young children and adults. 9
Correlation analysis for demographic covariates 11
Bivariate analysis 12
Correlations with demographic variables tended to be flat except median household size and median age where non-linear positive and inverse relationships with malaria positives were observed 12
Clusters in the South-East, North-West and South-West were at higher risk of malaria than those in the North-Central 15
Increases in the percentage of male agricultural workers correlated with small increases in malaria transmission intensity 17
Behavioral variables 18
Descriptive analysis 18
While bednet use was low, higher levels of medical health seeking was reported in constrast to the low receipt of effective fever treatment 18
Correlation analysis for behavioral variables 21
Bivariate analysis 22

Absence of significant visual relationship with behavioral variables, and differences in net use impacts by region 22
Accessibility factors 25
Descriptive analysis 25
Health care was highly accessible in most urban areas 25
Bivariate analysis 25
Environmental factors 26
Descriptive analysis 26
Surveys months fell within the transmission season for the South-West, South-East and South-South geopolitical zones and temperature was mostly uniform across all clusters at the time of the survey 26
Correlation matrix for environmental covariates 28
Bivariate analysis 29
Temperature maximally increases malaria transmission at around 30 ^{0}C while increases in the levels of enhanced vegetation index was positively correlated with small increases in transmission 29
Model diagnostics plots 32
Supplementary Table S1: Multivariable regression summary results for fixed effects 33
References 34

Test positivity rate by urban cluster, month and year of survey

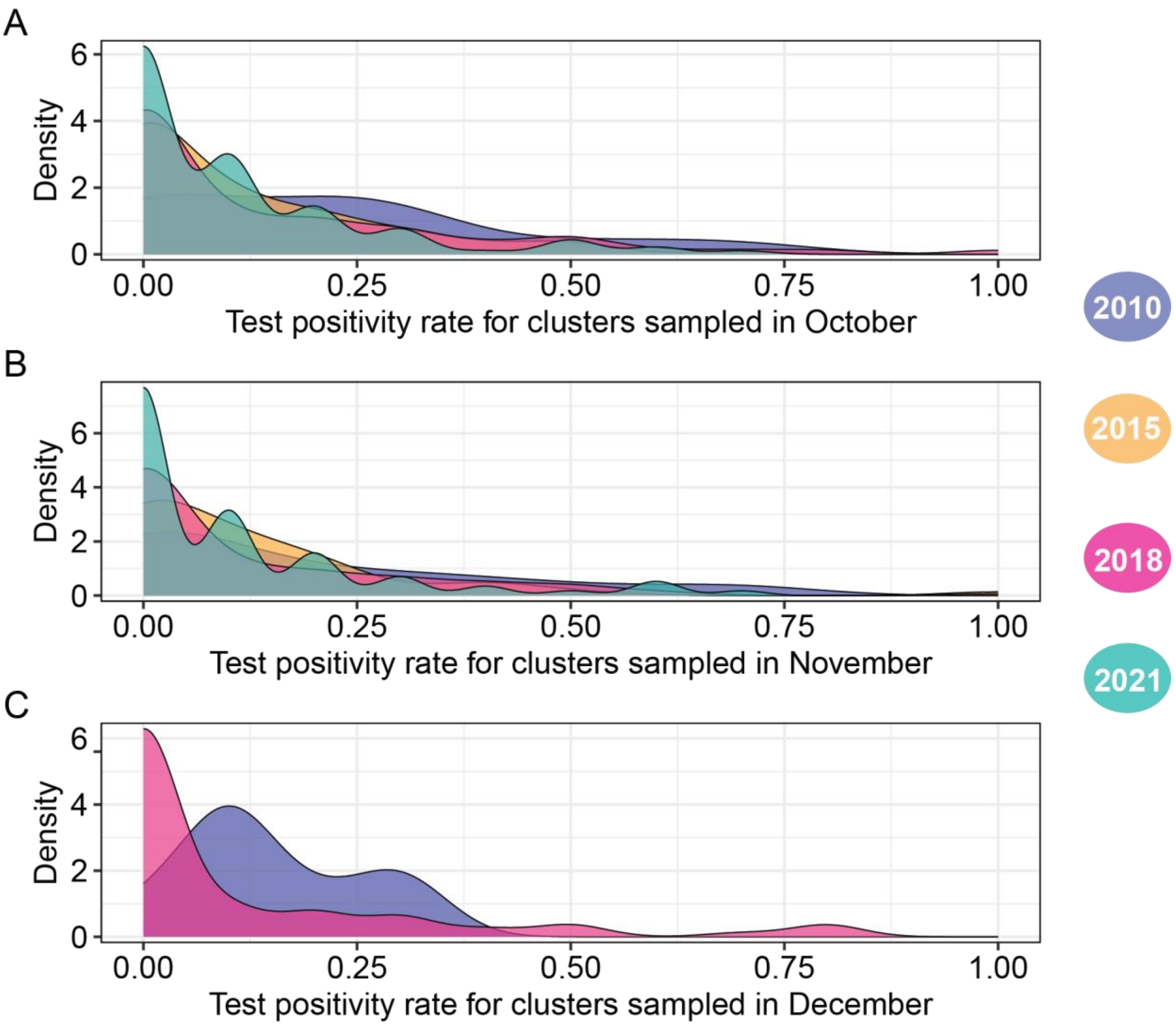

Supplementary Figure No: 1. Distribution of test positivity rates by DHS survey year and month. Plots are colored by survey year. The number of children 6 – 59 tested across all clusters and survey years was 4473 in October (A), 3352 in November(B), and 652 in December (C). The y-axis represents the probability density function from the kernel density estimation.

Socioeconomic factors

Descriptive analysis

Sampled clusters had low levels of post-primary education, fell within the rich wealth quintiles and had improved housing conditions

Within 52% of the 988 cluster samples, 13% or fewer individuals had post-primary education. The maximum percentage of persons with post-primary education in an individual cluster was 100% (Supplementary Figure No: 2a). The distribution of the wealth quintile and housing-related variable (improved flooring, homes with metal or zinc roof, and improved wall type) were similarly left-skewed (Supplementary Figure No: 2b – 2e), reflecting that wealth quintiles were constructed in the DHS using information on housing conditions.

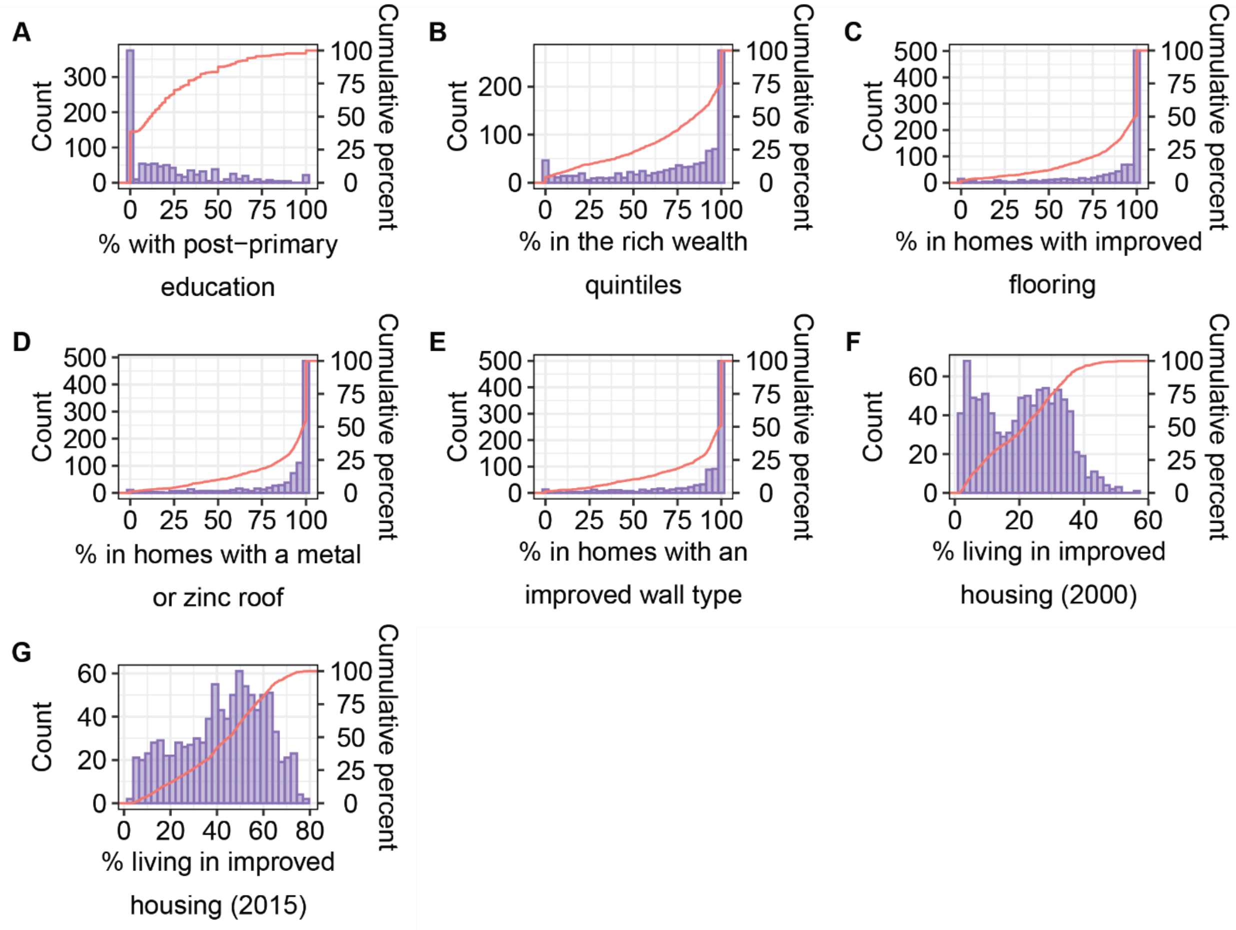

Supplementary Figure No: 2A – 2G. Distribution of socioeconomic covariates for all cluster samples. Each Supplementary Figure has two distribution illustrations and two y-axis. The purple histogram is each variable's frequency distribution while the red line is their cumulative distribution. The left y-axis corresponds to the number count for the purple histogram and the right y-axis corresponds to the cumulative percentage for the red line. A) Percentage of individuals with post-primary education (secondary or higher education). B) Percentage of individuals in the rich wealth quintiles. C) Percentage of individuals living in homes with improved flooring. D) Percentage of individuals living in homes with a metal or zinc roof. E) Percentage of individuals living in homes with an improved wall type. F) Percentage of individuals living in improved housing in year 2000. E) Percentage of individuals living in improved housing in year 2015.

In 85% and 99% of clusters, more than half of the inhabitants were in the rich quintiles or lived in homes with improved flooring, respectively. In addition, over half of cluster residents in 90% and 91% of clusters resided in homes with either a metal or zinc roof or with an improved wall type. The median improved housing coverage in 2000 and 2015 was 22% and 42%, respectively (Supplementary Figure No: 2f and 2g).

Correlation analysis for socioeconomic covariates

Strong positive correlations were observed between socioeconomic features. The percentage of individuals in the rich wealth quintiles had a 70% correlation with the percent of individuals living in homes with improved flooring or improved wall type (Supplementary Figure No: 3). The prevalence of improved housing in 2000 was 90% correlated with the prevalence of improved housing in 2015.

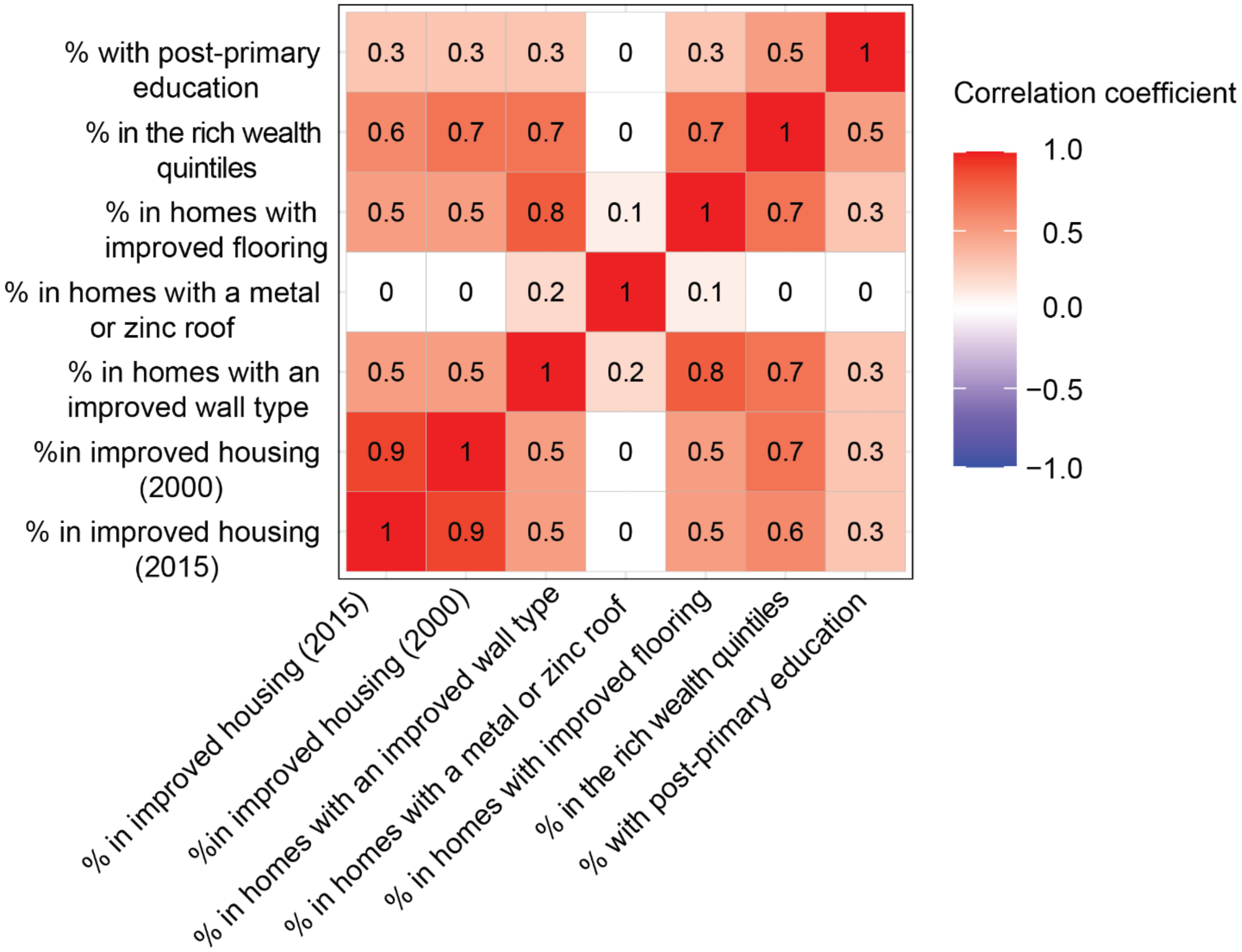

Supplementary Figure No: 3. Correlation matrix for socioeconomic covariates. Numeric values in each box are Pearson's correlation coefficient. Percentage of individuals in the rich or richest wealth quintiles had a strong positive correlation to the percent of individuals living in homes with improved wall type or flooring. The prevalence of improved housing in 2000 was 90% correlated with prevalence of improved housing in 2015.

Bivariate analysis

For most indicators of socioeconomic status, the lowest values correlated with higher levels of malaria positivity and test positivity rates

Aside from improved flooring indicator, the number of malaria positives among children 6 – 59 months predicted by the Poisson model were greatest at the lowest values of each socioeconomic indicator

(Supplementary Figure No: 4). Increases in the percent of individuals with post-primary education per cluster was correlated with declines in the number of malaria positives (Supplementary Figure No: 4a). Since the numbers of positive malaria tests, even at low levels of educational attainment, were small, the predicted trend flattens out at around 50% on the x-axis.

Declines in malaria positives with increasing percentage values of individuals in the rich wealth quintiles was fairly stable up to 25% before flattening,followed by additional declines at around 67% of individuals in the rich wealth quintiles (Supplementary Figure No: 4b). Surprisingly, as the percentage of homes with metal or zinc iron roof increased, the number of malaria positives increased before declines were observed between 50% and 75% (Supplementary Figure No: 4c). Similar to the wealth quintile indicator, the predicted trend for the individual correlations between the number of malaria positives, the percentage of homes with a improved flooring and the percentage of homes with an improved wall type was highest at the lower end of the horizontal scale followed by a period of decline, a subsequent bump, and additional declines at the highest predictor values (Supplementary Figure No: 4d – e). The bivariate relationship between improved housing variables and the number of malaria positives were mostly flat, although the maximum number of malaria positives were predicted to occur at the lowest values of these covariates (Supplementary Figure No: 4f – g). Additional analysis of bivariate associations of malaria test positivity rates with socioeconomic indicators in a Quasipoission model revealed similar trends, but the fitted lines for improved housing indicators were less flat (Supplementary Figure No: 4).

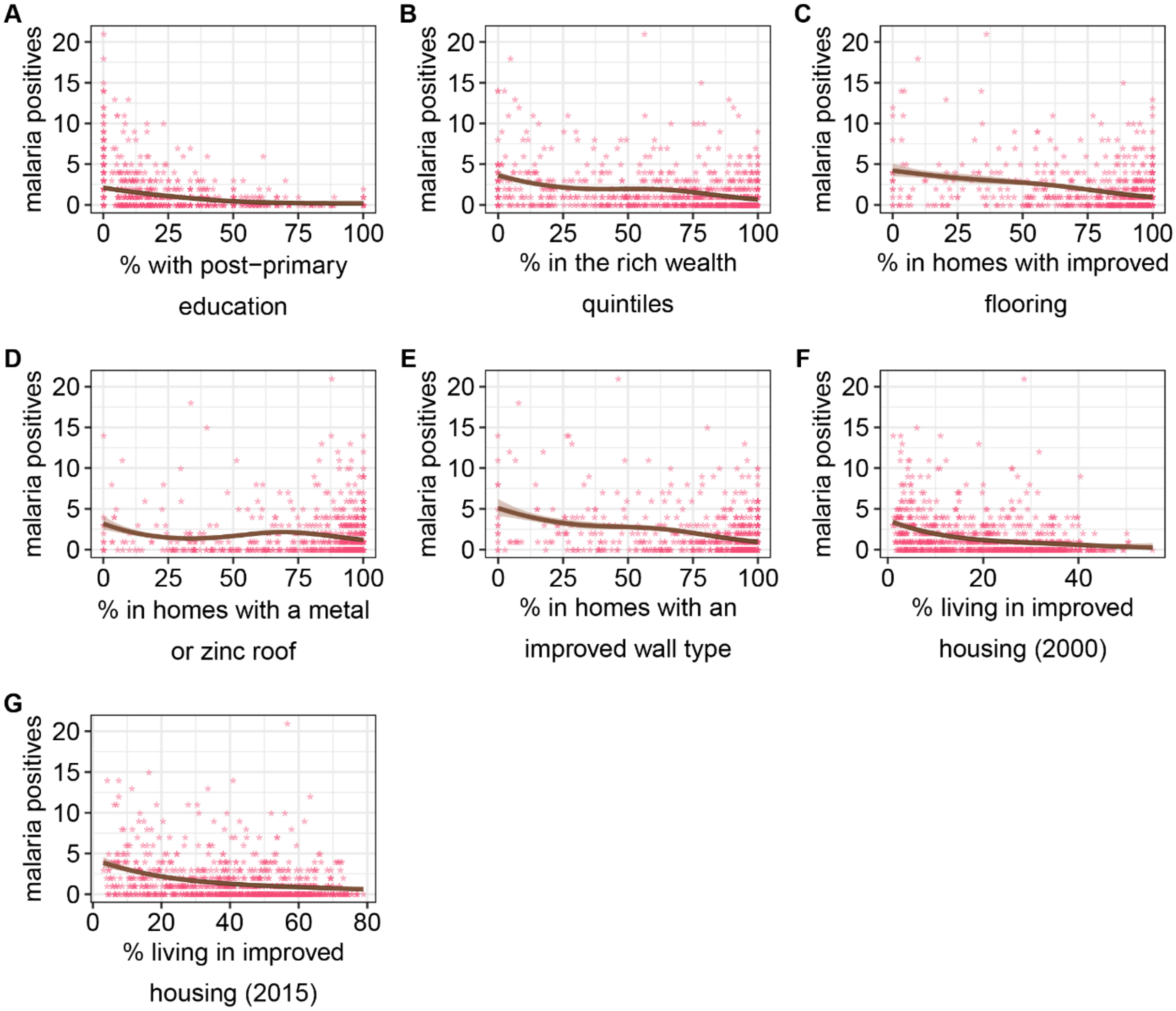

Supplementary Figure No: 4A - 4G. Bivariate associations between the number of malaria positives per cluster and socioeconomic covariates. Red stars are cluster data points. Brown lines and shaded areas are the Poisson regression model fit and 95% confidence intervals respectively. A) Percentage of individuals with post-primary education (secondary or higher education). B) Percentage of individuals in the rich or richest wealth quintiles. C) Percentage of individuals living in homes with improved flooring. D) Percentage of individuals living in homes with a metal or zinc roof. E) Percentage of individuals living in homes with an improved wall type. F) Percentage of individuals living in improved housing in year 2000. G) Percentage of individuals living in improved housing in year 2015.

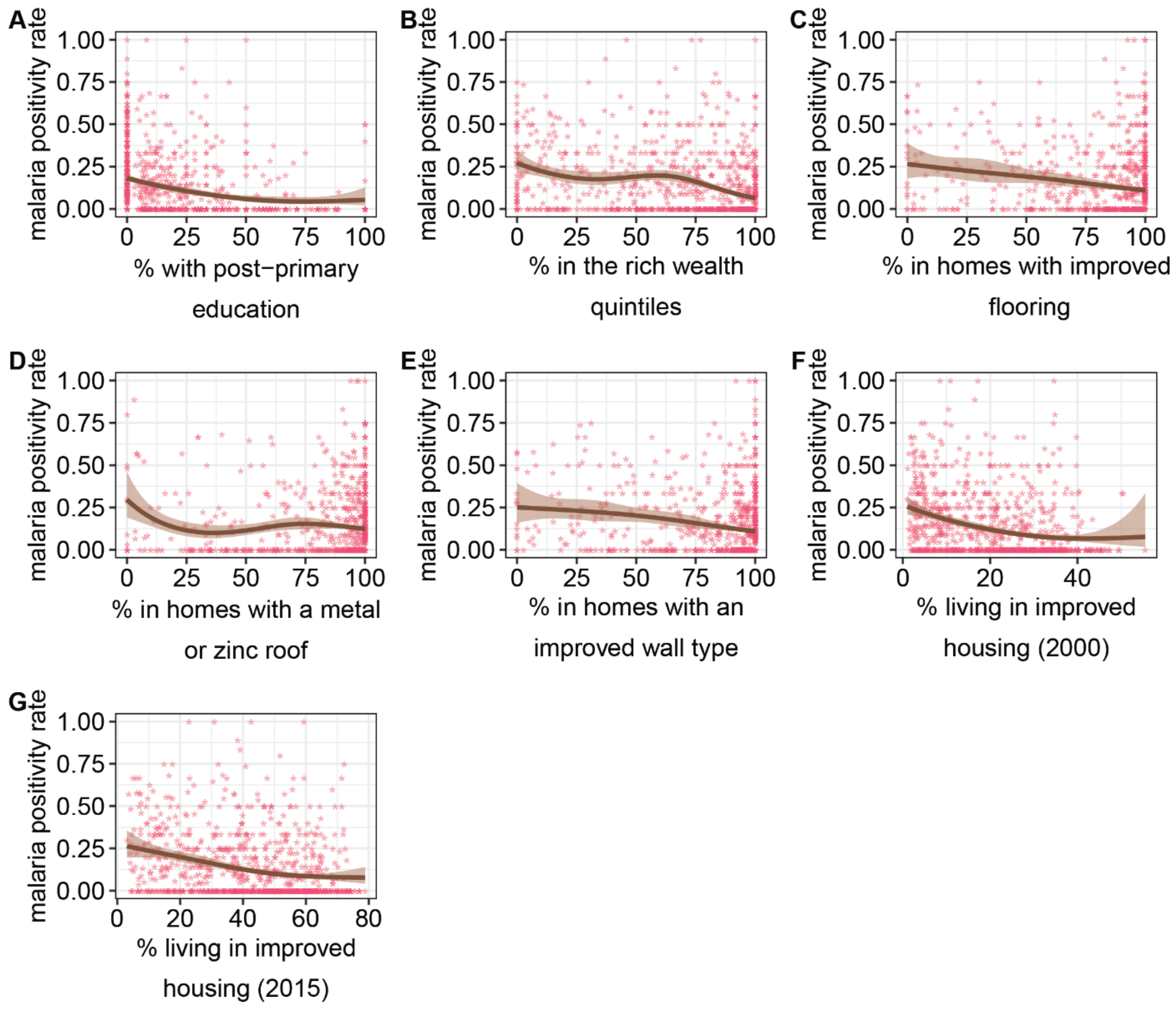

Supplementary Figure No: 5A - 5G. Bivariate associations between malaria test positivity rate and socio-economic covariates. Red stars are cluster data points. Brown lines and shaded areas are a Quasipoisson regression model fit and 95% confidence intervals respectively. A) Percentage of individuals with post-primary education (secondary or higher education). B) Percentage of individuals in the rich or richest wealth quintiles. C) Percentage of individuals living in homes with improved flooring. D) Percentage of individuals living in homes with a metal or zinc roof. E) Percentage of individuals living in homes with an improved wall type. F) Percentage of individuals living in improved housing in year 2000. E) Percentage of individuals living in improved housing in year 2015.

Demographic variables

Descriptive analysis

Cluster populations were generally young children and adults.

Population density values varied widely from 25 to 35,029 persons per square kilometer. 50% and 75% of clusters had a population density estimate at or below 746 and 2081 persons per square kilometers, respectively (Supplementary Figure No: 6a). Per cluster population densities for children under the age of five years were also widely dispersed ranging from 0.30 to 215 persons per square kilometer (Supplementary Figure No: 6b). Thirty-five percent of clusters had no pregnant women (Supplementary Figure No: 6c). The median household size ranged from 2 to 14, with 50% of clusters reporting a household size of 5 or fewer persons (Supplementary Figure No: 6e). Clusters were mostly populated by young persons. The median age ranged from 10 to 44 years, and 50% of clusters had a median age of 19 years or less (Supplementary Figure No: 6f). The number of sampled clusters varied by state. Lagos state had the largest number of sampled clusters at 81, followed by Anambra and Oyo with and 50 clusters each, reflecting large population sizes and the need to capture associated heterogeneities in the DHS indicators (Supplementary Figure No: 6g) [1]. The South-West, South-East and North-Central geopolitical zones had the largest numbers of sampled clusters at 281, 171, and 159, respectively (Supplementary Figure No: 6h).

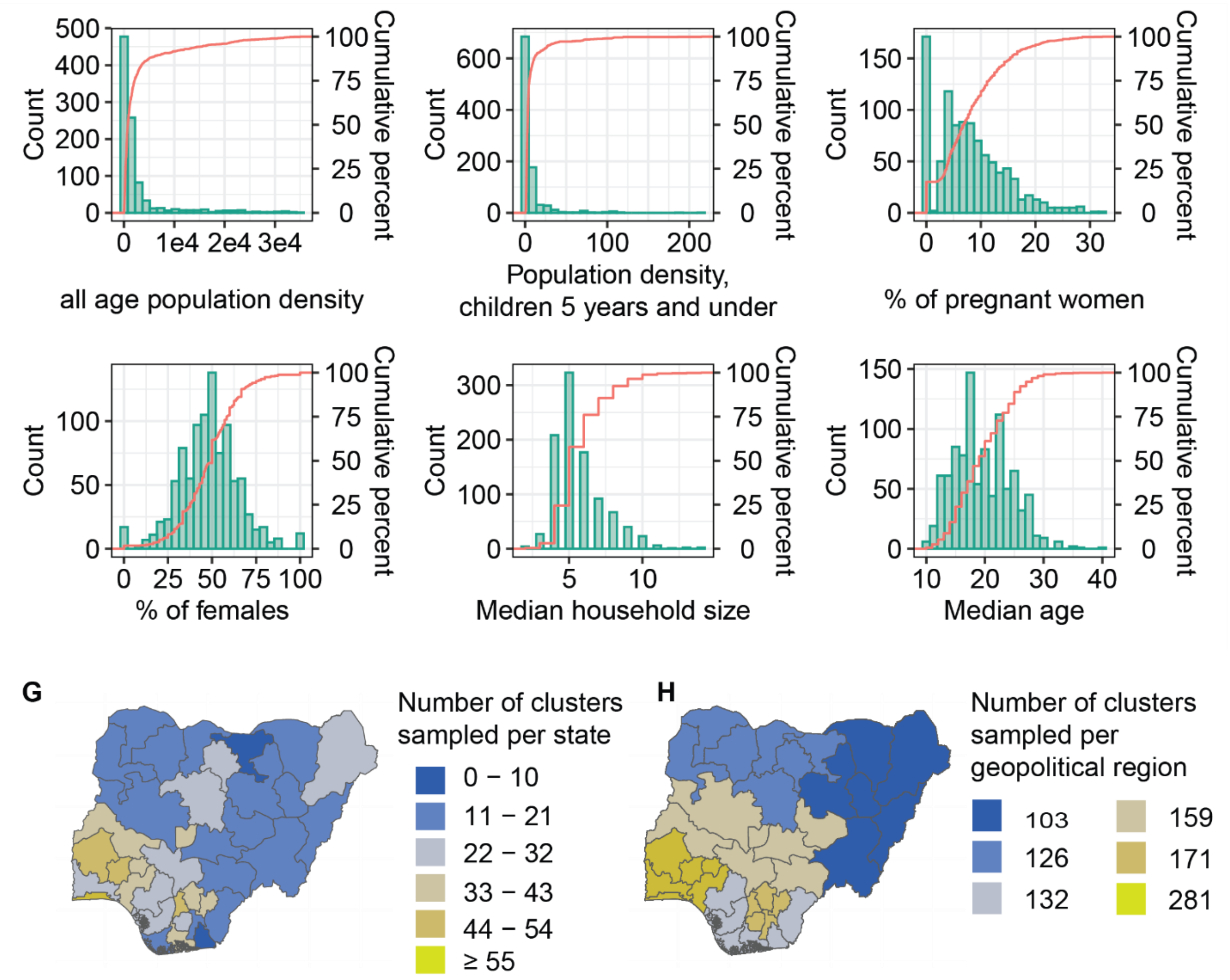

Supplementary Figure No: 6A – 6G. Distribution of demographic covariates for all cluster samples. Supplementary Figure No: 6A-F has two distribution illustrations and two y-axes. The green histogram is each variable's frequency distribution while the red line is their cumulative distribution. The left y-axis corresponds to the number count for the green histogram and the right y-axis corresponds to the cumulative percentage for the red line. A) All age population density (persons per square kilometer). B) Population density among children 5 years and under (persons per square kilometer). C) Percentage of pregnant women. D) Percentage of females per cluster. E) Median household size. F) Median age. G) Number of clusters sampled per state. H) Number of clusters sampled per geopolitical region.

Correlation analysis for demographic covariates

Median household size had a 60% negative linear relationship with median age (Supplementary Figure No: 7).

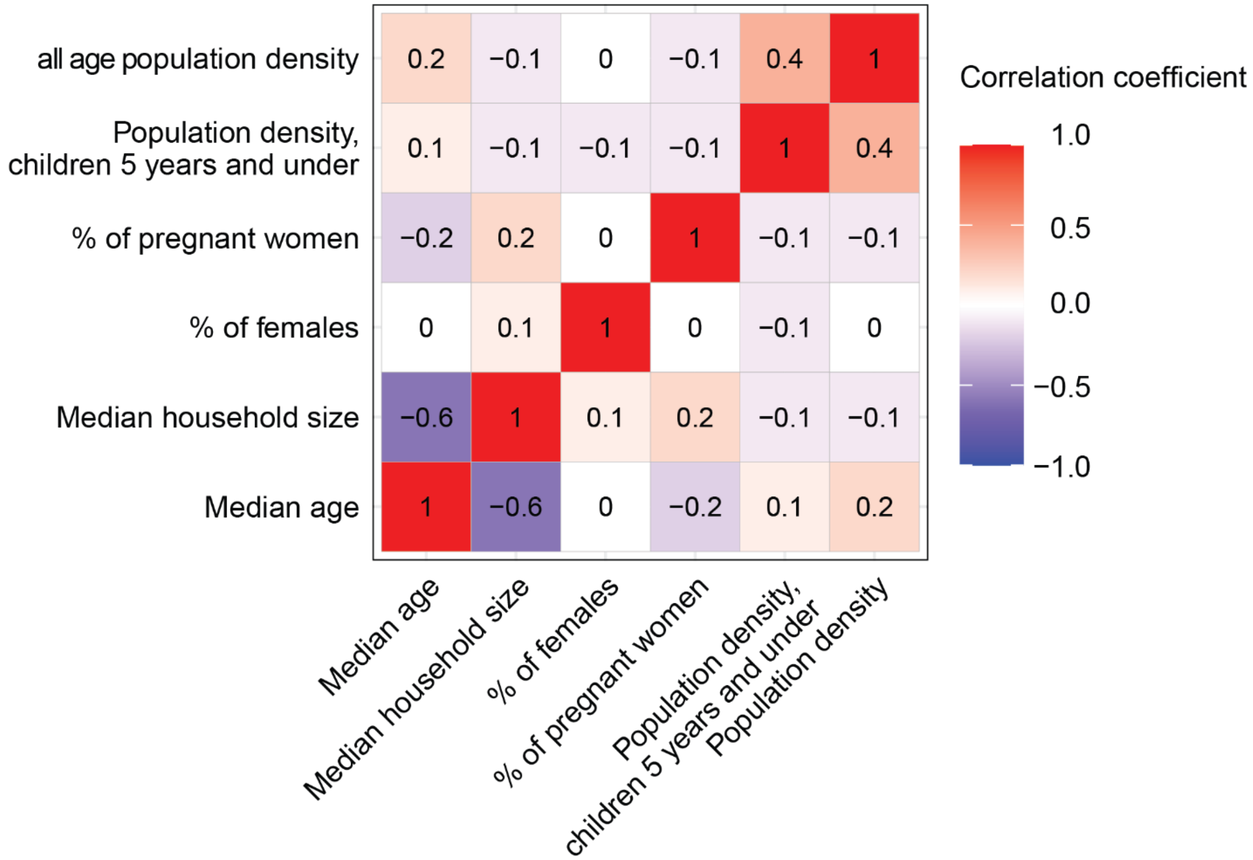

Supplementary Figure No: 7. Correlation matrix for demographic covariates. Numeric values in each box are Pearson's correlation coefficient. Median household size had a strong negative correlation of 60% with median age.

Bivariate analysis

Correlations with demographic variables tended to be flat except median household size and median age where non-linear positive and inverse relationships with malaria positives were observed

The fitted lines for all age population density, under-five population density, the percentage of pregnant women and the percentage of females per cluster indicates a mostly uniform relationship with the number of malaria positives among children 6 – 59 months, although it tended to exhibit a fairly positive linear relationship to the left of the horizontal scale (Supplementary Figure No: 8a – d). While this may be an artefact of the data, it might also suggest that specific ranges of these covariates (20 – 395 persons per square kilometer for all age population density, 1.8 – 4.9 persons per square kilometer for under-five population density, 0 – 16% for percent of pregnant women, and 0 – 48% for the percent of females) are associated with higher numbers of malaria positives in children. Increases in the number of malaria positives followed an increase in median household sizes peaking at a median household size of 8 before declines, likely due to data sparsity, were observed (Supplementary Figure No: 8e – f). Increases in median age was correlated with declines in the number of malaria positives among children. Higher values of median age from 10 – 15 years was associated with a higher number of malaria positives and had a prominent slope, indicating that transmission is concentrated within clusters with the most numbers of children. Since median household size is 60% negative correlated with age, it implies that larger households may have more children which may explain the bump in the number of malaria positives at household sizes between 5 and 10. Extreme values for population density (> 3000), under-five population density (> 10) , median household size (>30), and median age (>30) were eliminated in Supplementary Figure No: 8 to enable visualization of trends at the lower ends of the horizontal scale where most data points are concentrated.

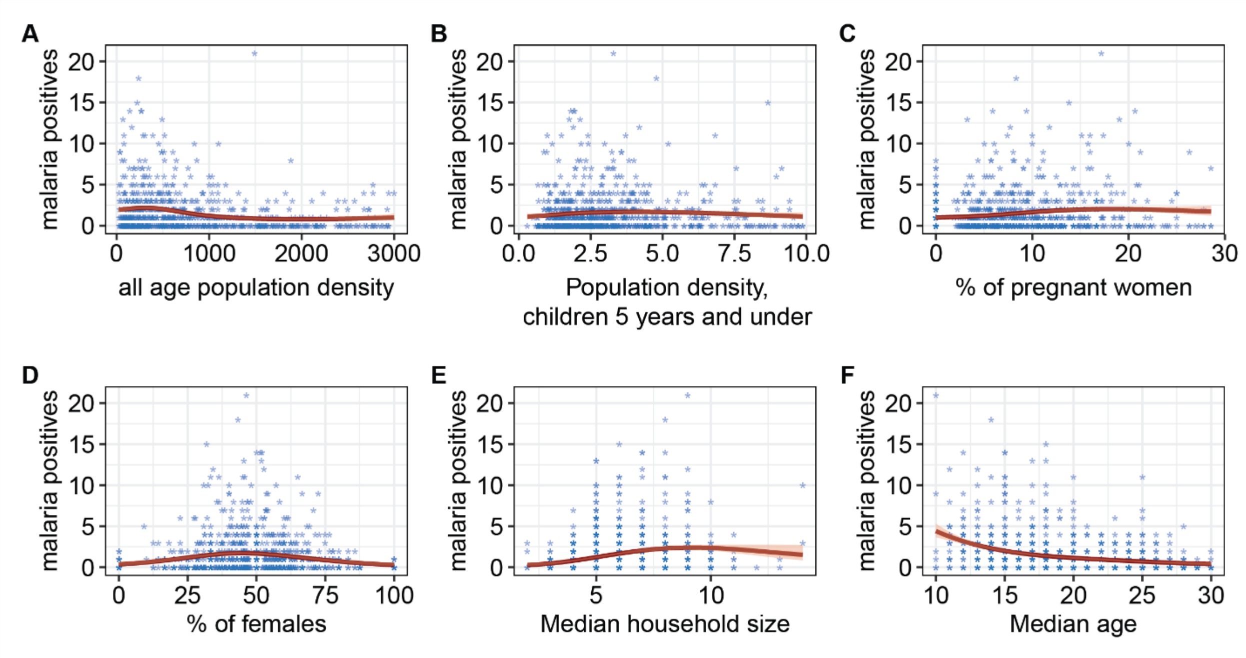

Supplementary Figure No: 8A - 8F. Bivariate associations between the number of malaria positives per cluster and demographic covariates. Blue stars are cluster data points. Dark-red lines and salmon shaded areas are the Poisson regression model fit and 95% confidence intervals respectively. A) Population density (persons per square kilometer). Due to right skewed distribution, plotted values are limited to ≤ 3000 to more clearly visualize bivariate associations with malaria positives. B) Population density among children 5 years and under (persons per square kilometer). Plotted values are limited to ≤ 10 due to right skewed distribution. C) Percentage of pregnant women. Plotted values are limited to ≤ 30 due to right skewed distribution. D) Percentage of females per cluster. E). Median household size. F) Median age. Plotted values are limited to ≤ 30 years due to right skewed distribution.

The effect of outlying values on the fitted line are evident for all age population density and the percent of pregnant women per cluster in Supplementary Figure No: 9. The highest values of the numbers of malaria positives were observed at the most extreme population density values while the lowest numbers of malaria positives occurred at very high percentages of pregnant women per cluster. Correlations between malaria test positivity rate and demographic variables in a quasipossion model are fairly similar, except for the percentage of pregnant women and the percent of females per cluster

where a roughly linear positive and negative relationship were respectively observed (Supplementary Figure No: 9).

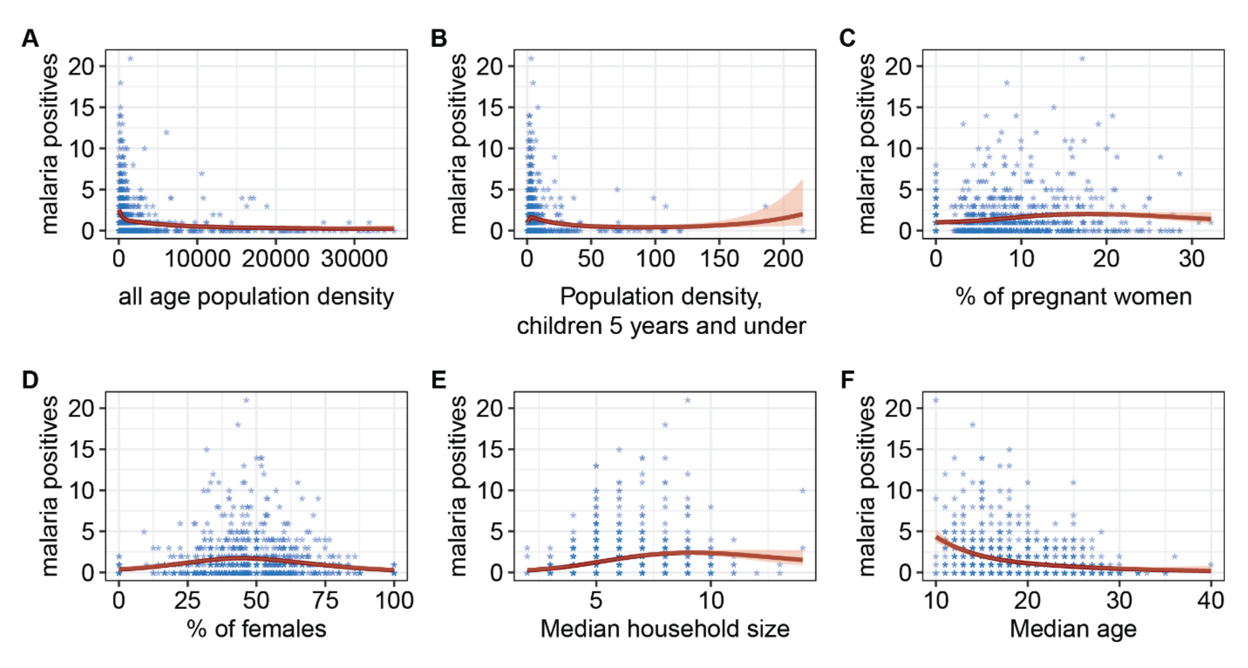

Supplementary Figure No: 9A – 8F. Bivariate association between the number of malaria positives per cluster and demographic covariates. Blue stars are cluster data points. Dark-red lines and salmon shaded areas are a Poisson regression model fit and 95% confidence intervals respectively. A) Population density (persons per square kilometer). B) Population density among children 5 years and under (persons per square kilometer. C) Percentage of pregnant women. D) Percentage of females per cluster. E). Median household size. F) Median age.

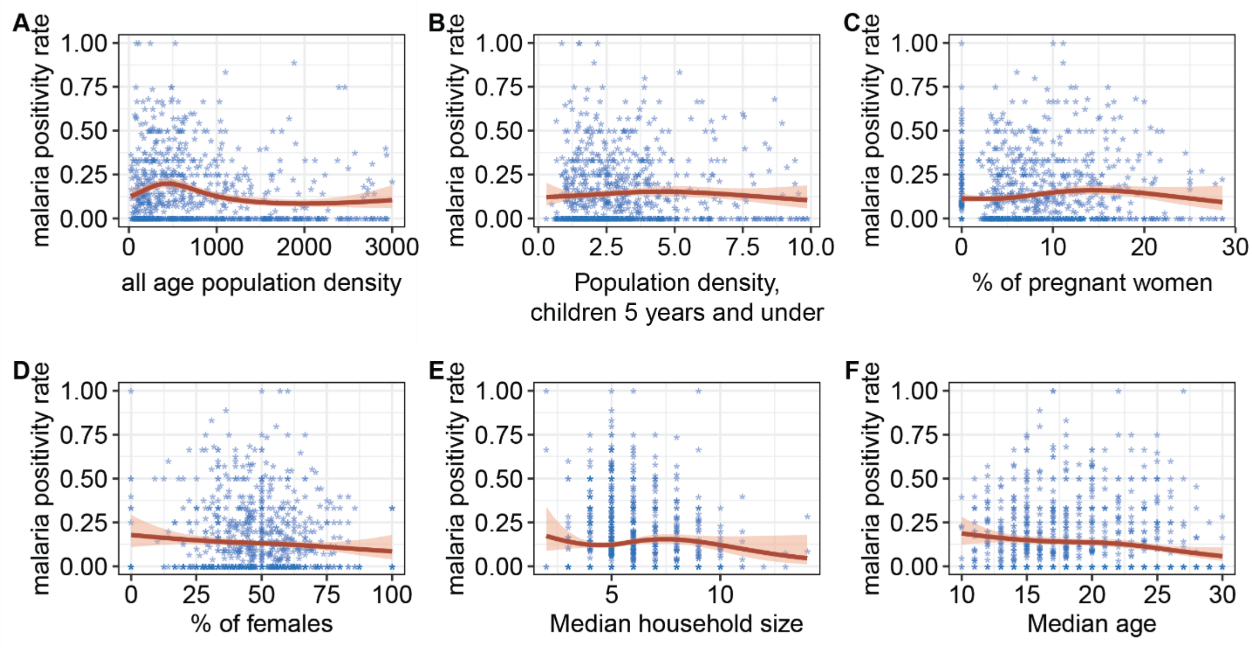

Supplementary Figure No: 10A – 10F. Bivariate association between the number of malaria positives per cluster and demographic covariates. Blue stars are cluster data points. Dark-red lines and salmon shaded areas are the Quasipoisson regression model fit and 95% confidence intervals respectively. A) Population density (persons per square kilometer). Due to right skewed distribution, plotted values are limited to ≤ 3000 to more clearly visualize bivariate associations with malaria positives. B) Population density among children 5 years and under (persons per square kilometer). Plotted values are limited to ≤ 10 due to right skewed distribution. C) Percentage of pregnant women. Plotted values are limited to ≤ 30 due to right skewed distribution. D) Percentage of females per cluster. E). Median household size. F) Median age. Plotted values are limited to ≤ 30 years due to right skewed distribution.

Clusters in the South-East, North-West and South-West were at higher risk of malaria than those in the North-Central

Comparisons of the slope estimates for individual states with that of Abia state (a lower risk malaria state) suggests that clusters in Ebonyi, Ekiti, Kebbi, Ondo, Osun, and Sokoto were more likely to report statistically significant larger numbers of malaria positives while 5 other states including Lagos and Plateau were significantly more likely to report fewer malaria positives (Supplementary Figure No: 11).

Similarly, compared to clusters in the North-central, clusters in the South-East, North-West and South-West had a higher likelihood of reporting more malaria positives (Supplementary Figure No: 11)

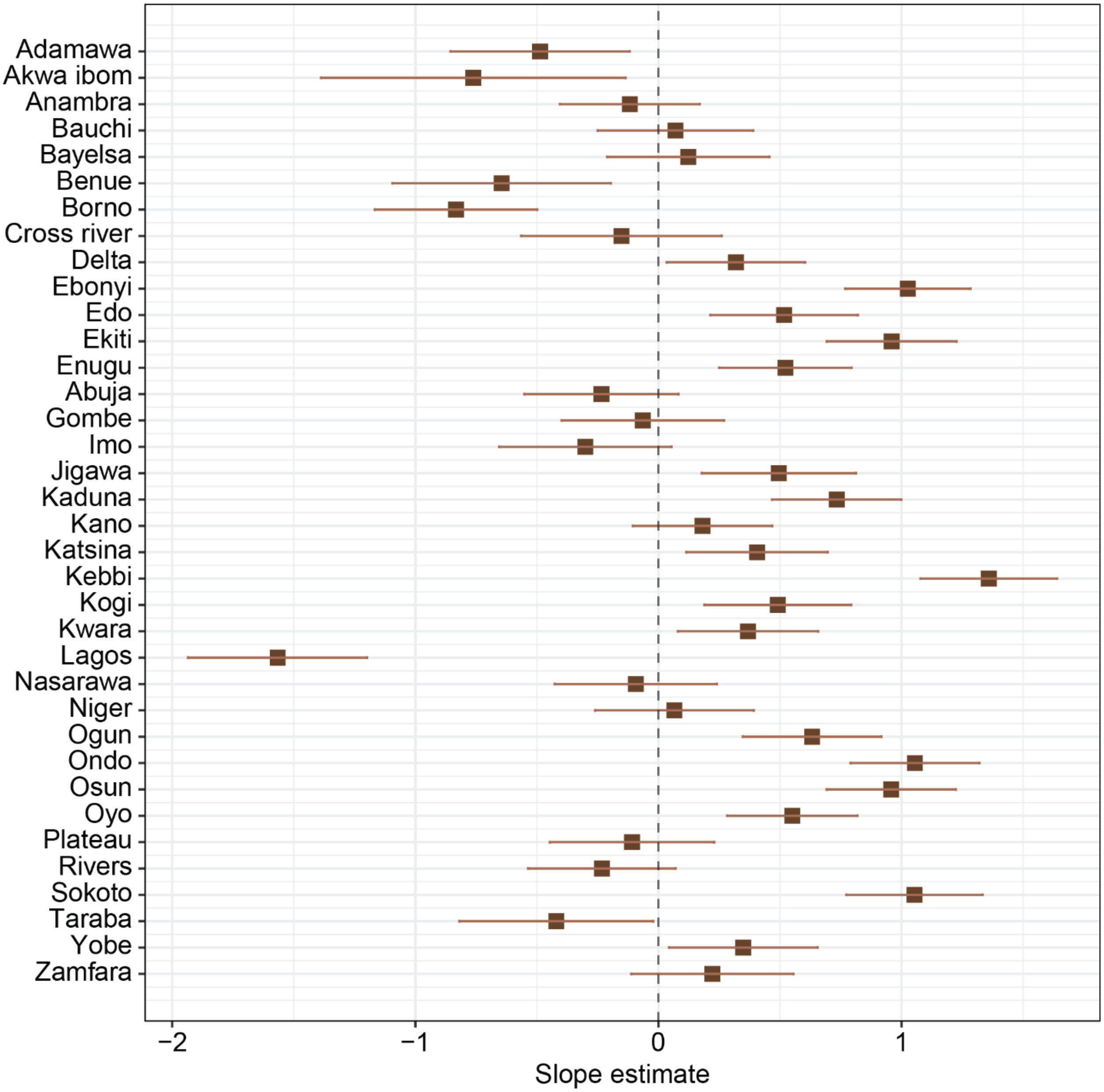

Supplementary Figure No: 11. Forest plot comparing Poisson regression model slope estimates for individual states using Abia state as the reference category. Negative slope estimates indicate a lower likelihood of a state having clusters with high numbers of malaria positives and vice-versa for positive slope estimates.

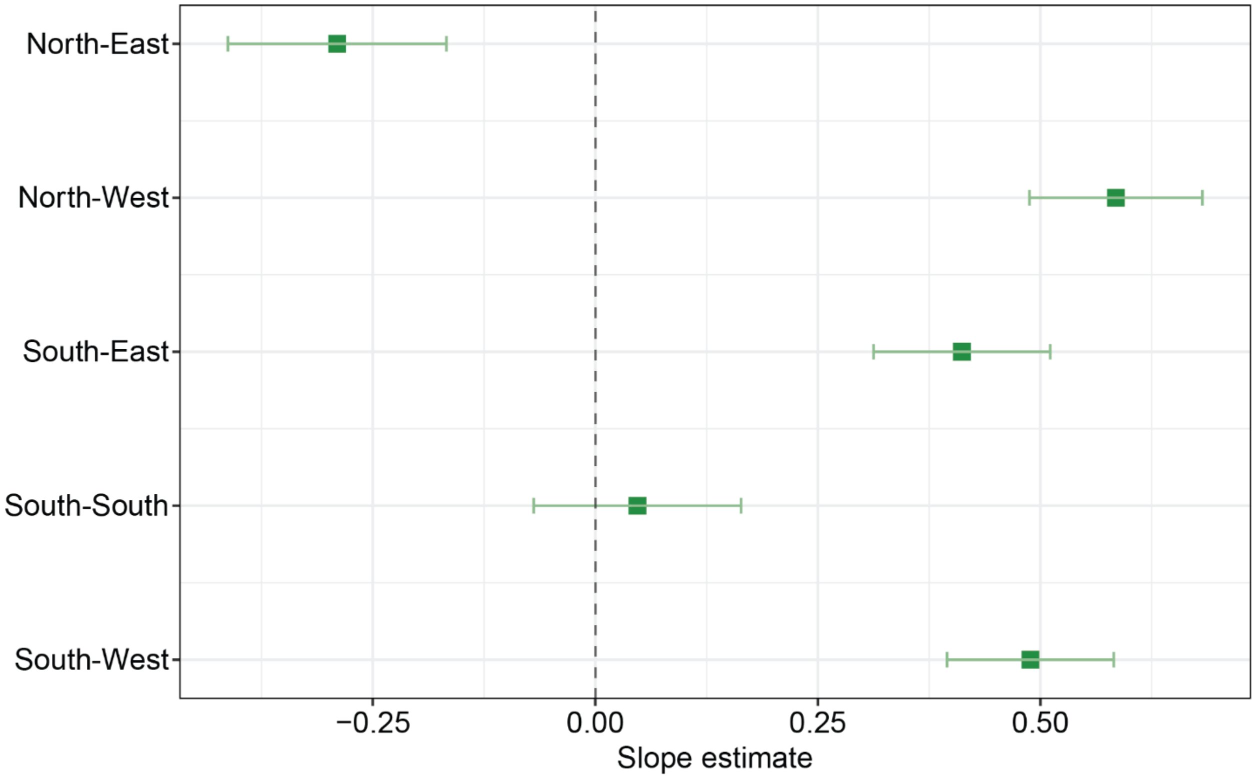

Supplementary Figure No: 12. Forest plot comparing Poisson regression model slope estimates for geopolitical zones using the North-Central geopolitical zone as the reference category. Negative slope estimates indicate a lower likelihood of a geopolitical zone having clusters with high numbers of malaria positives and vice-versa for positive slope estimates.

Increases in the percentage of male agricultural workers correlated with small increases in malaria transmission intensity

Supplementary Figure No: 13 depicts the relationship between the numbers of malaria positives and occupation-related variables, which are only available in the 2018 DHS. Bivariate associations between malaria positives and the percentage of male partners that are agricultural workers is curvilinear, increasing linearly up to around 50% before declining. The observed curvilinear relationship may be due to data sparsity at the higher extremes of the covariate. The relationship with the percentage of male

seasonal workers is mostly flat, although it exhbits a slight curvilinear form. Additionally, the bivariate association with the percentage of female seasonal workers is flat and exhbits high levels of uncertainity at the higher extremes.

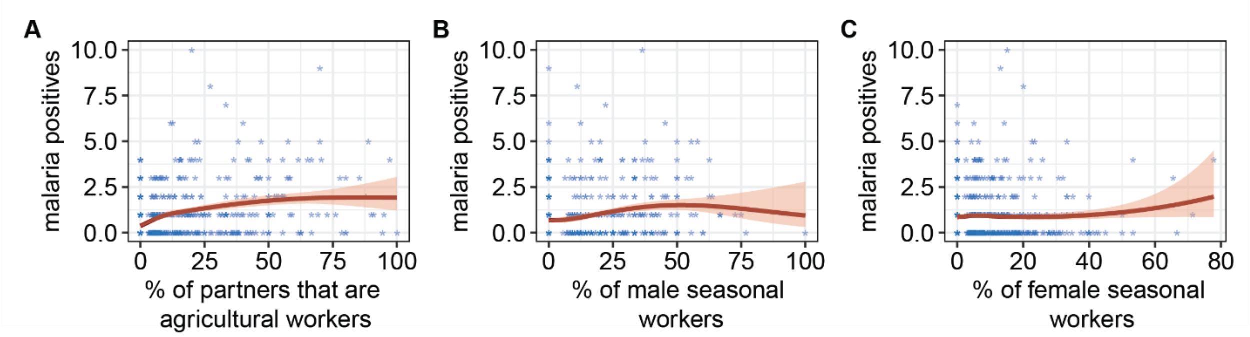

Supplementary Figure No: 13A – 13C. Bivariate association between the number of malaria positives per cluster and occupation-related covariates from the 2018 Demographic and Health Survey. Dark-red lines and salmon shaded areas are a Poisson regression model fit and 95% confidence intervals respectively. A) Percentage of male partners that are agricultural workers. B) Percentage of male seasonal workers. C) Percentage of female seasonal workers.

Behavioral variables

<u>Descriptive analysis</u>

<u>While bednet use was low, higher levels of medical health seeking was reported in constrast to the low receipt of effective fever treatment</u>

The median percentage of individuals using a bednet per cluster was 28% (IQR: 29%) (Supplementary Figure No: 14a), with only 11 (1.1%) of 972 surveyed clusters achieving 80% or greater target utilization value for malaria preventive measures specified in the 2009 – 2013 National Malaria Strategic Plan (NMSP) [2]. However, among those have access to ITN, the median usage per cluster was higher at 74% (IQR: 43%) (Supplementary Figure No: 14). Similarly, the percentage of children 6 - 59 months that use a bednet for sleeping, among those tested for malaria using microscopy, was low. Only 106 10.9%) of the

972 clusters achieved NMSP usage target values (Supplementary Figure No: 14b). In nearly 32% of 830 clusters with available data for analysis, 100% of children under the age of five years received medical treatment for fever and the median cluster value for medical treatment seeking given fever was 67% (Supplementary Figure No: 14c). Whereas, in 43% of 658 clusters, no child under the age of five years with fever received an artemisinin-based combination therapy (effective fever treatment) and the median value was 4% (Supplementary Figure No: 14d).

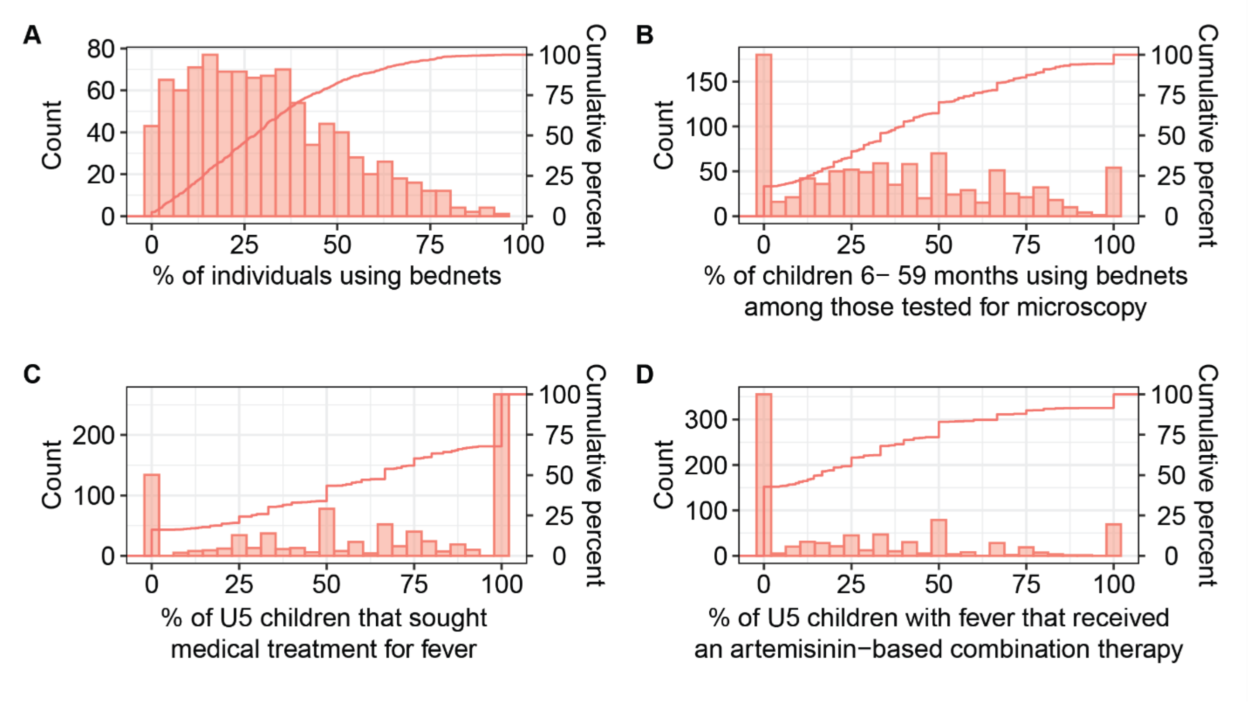

Supplementary Figure No: 14A - 14D. Distribution of behavioral covariates for all cluster samples. Each Supplementary Figure has two distribution illustrations and two y-axes. The salmon-colored histogram is each variable's frequency distribution while the red line is their cumulative distribution. The left y-axis corresponds to the number count for the salmon-colored histogram and the right y-axis corresponds to the cumulative percentage for the red line. A) Percentage of individuals using bednets. B) Percentage of children 6 – 59 months using bednets among those tested for malaria using a microscopy. C) Percentage of children under five years that sought medical treatment for fever. D) Percentage of children under five years with fever that received an artemisinin combination therapy.

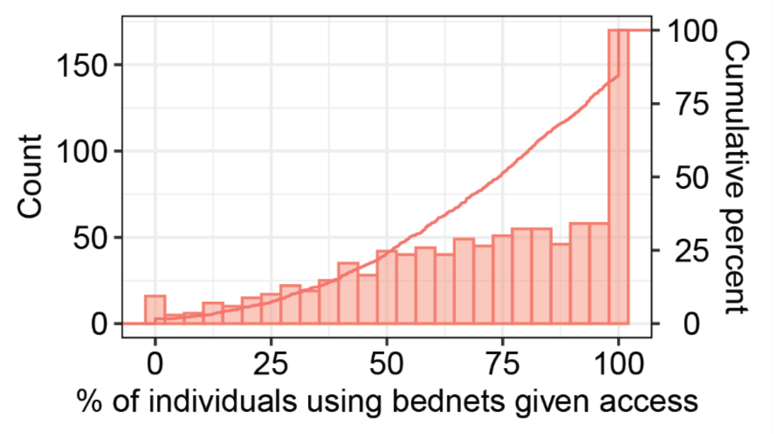

Supplementary Figure No: 15. Distribution plot for the percent of individuals that use bednets given that they have access. The Supplementary Figure has two distribution illustrations and two y-axis. The salmon-colored histogram is the frequency distribution while the red line is the cumulative distribution. The left y-axis corresponds to the number count for the salmon-colored histogram and the right y-axis corresponds to the cumulative percentage for the red line.

Correlation analysis for behavioral variables

A 70% positive linear correlation was observed between the percent of individuals using bednets per cluster and net use among children 6 –59 months who were tested for malaria (Supplementary Figure No: 16).

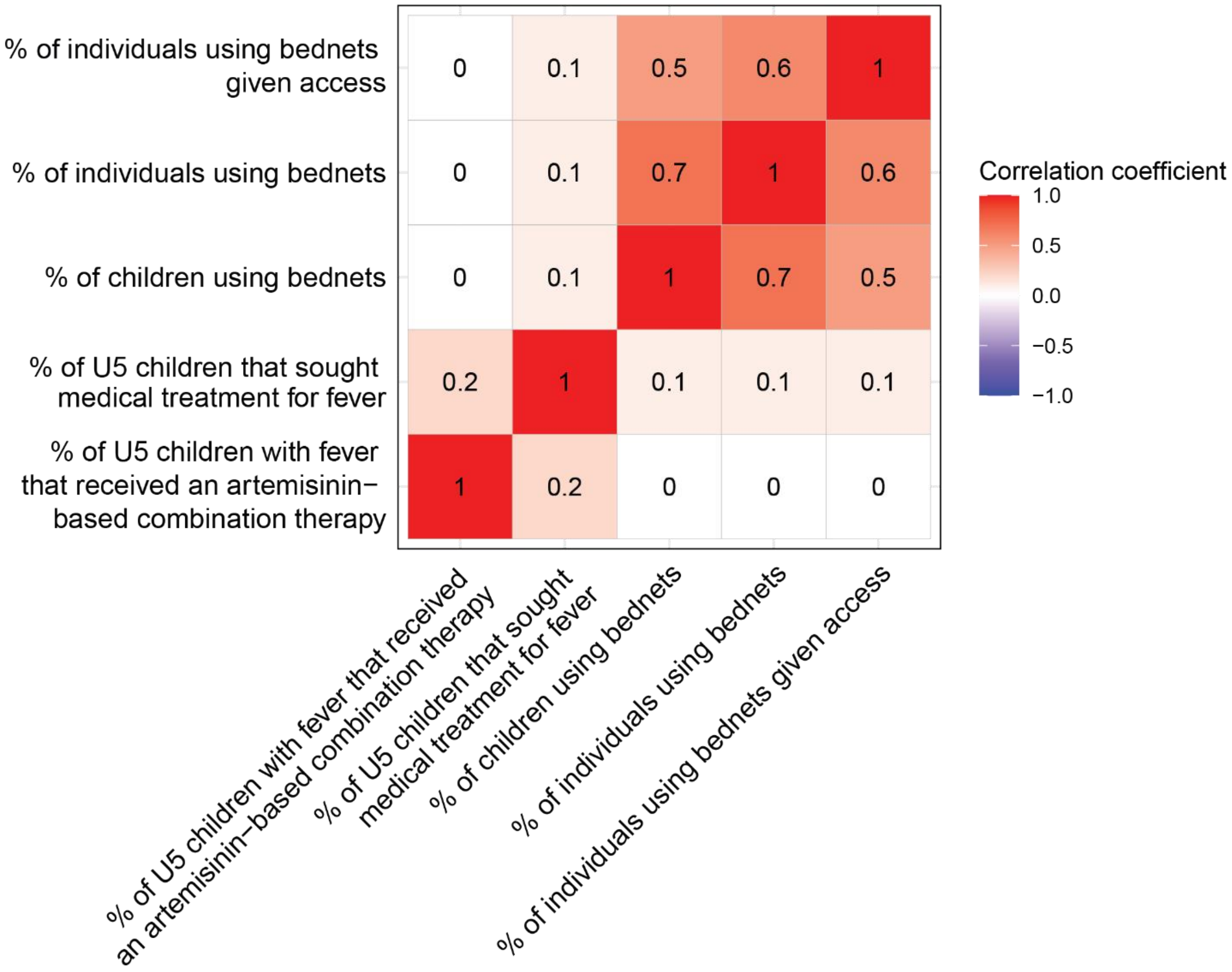

Supplementary Figure No: 16. Correlation matrix for behavioral covariates. Numeric values in each box are Pearson’s correlation coefficient. Overall cluster nets had a strong positive correlation of 70% with net use in children 6 – 59 months.

Bivariate analysis

Absence of significant visual relationship with behavioral variables, and differences in net use impacts by region

The fitted line for all behavioral variables were mostly flat (Supplementary Figure No: 17 and 18). Stratified analysis by geopolitical region, shows an elevated number of malaria positives at very low-levels of net use in three of the six regions, with the highest impact in the North-West region (Supplementary Figure No: 18). Counterintuitive findings such as the slight bump in the number of malaria positives between 50 – 75% net use values in Supplementary Figure No: 17a and the slight curvilinear relationship with the percentage of U5 children that sought medical treatment for fever, is likely due to the effects of confounding and/or poor data representativeness.

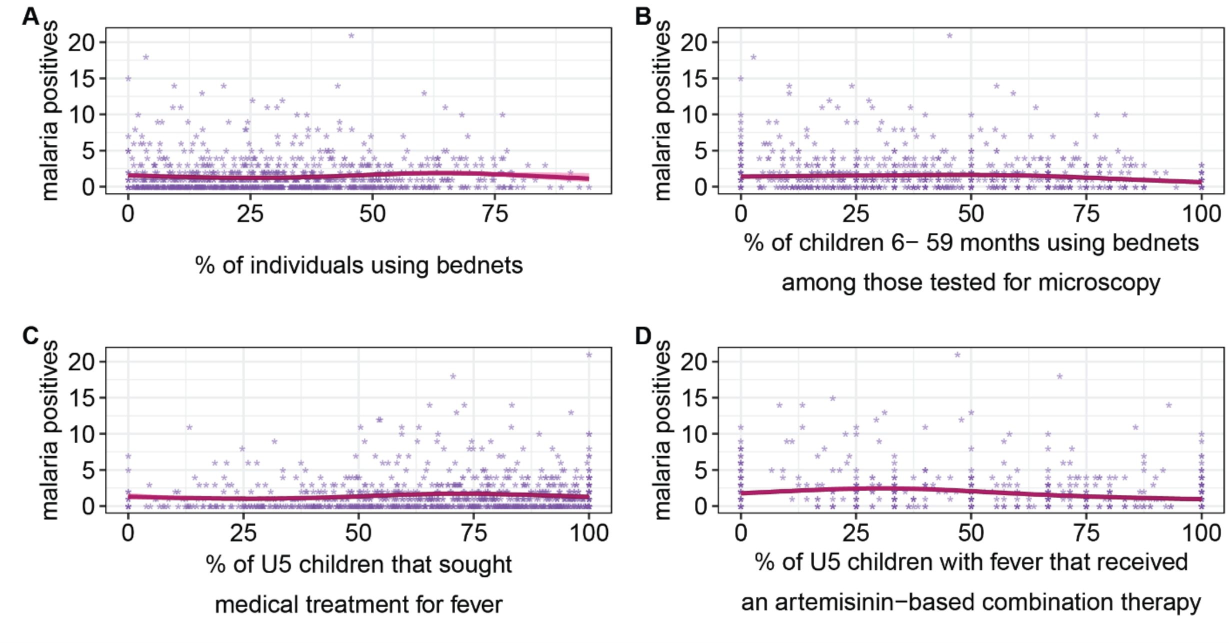

Supplementary Figure No: 17A – 17D. Bivariate association between the number of malaria positives per cluster and behavioral covariates. Purple stars are cluster data points. Deep pink lines and shaded areas are the Poisson regression model fit and 95% confidence intervals respectively. A) Percentage of indviduals using bed nets (Bed net use is defined as those that slept under a treated net the night before the survey). B). Percentage of children 6 – 59

months using bednets among those tested for malaria. C) Percentage of children under the age of five years that sought medical treatment for fever. D) Percentage of children under the age of five with fever that received an ACT.

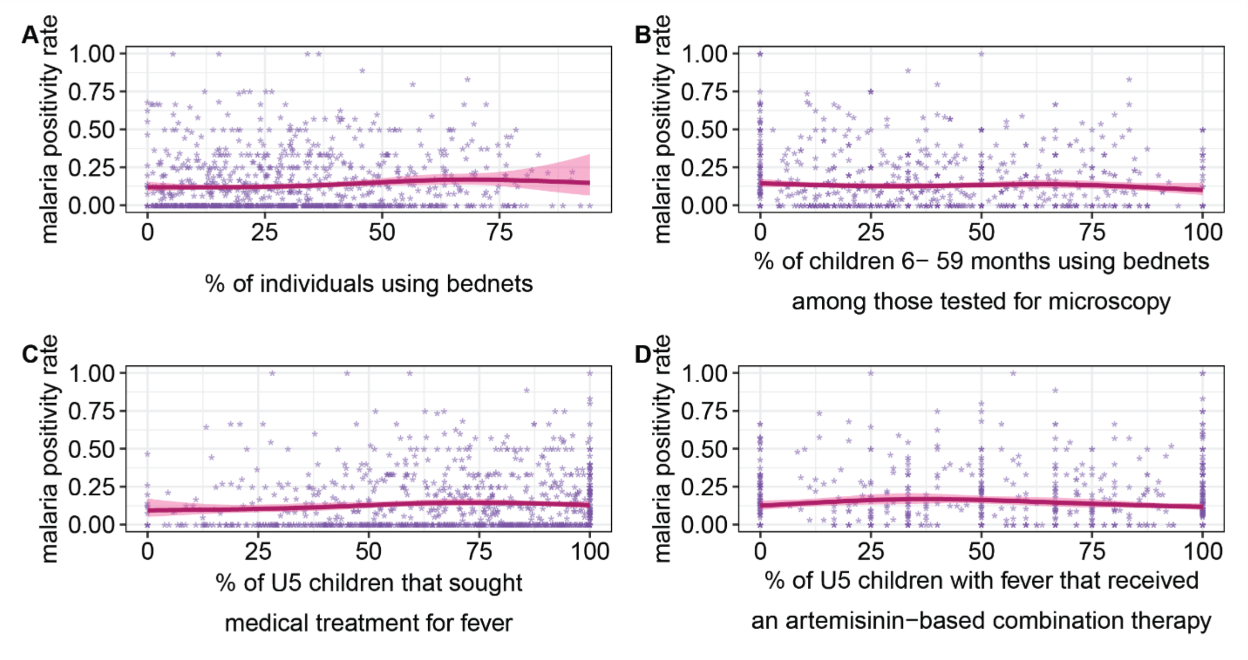

Supplementary Figure No: 18A – 18D. Bivariate associations between malaria test positivity rate and behavioral covariates. Purple stars are cluster data points. Deep pink lines and shaded areas are a Quasipoisson regression model fit and 95% confidence intervals respectively. A) Percentage of individuals using bed nets (Bed net use is defined as those that slept under a net the night before the survey). B) Percentage of children using bed nets. C) Percentage of children under the age of five years that sought medical treatment for fever.

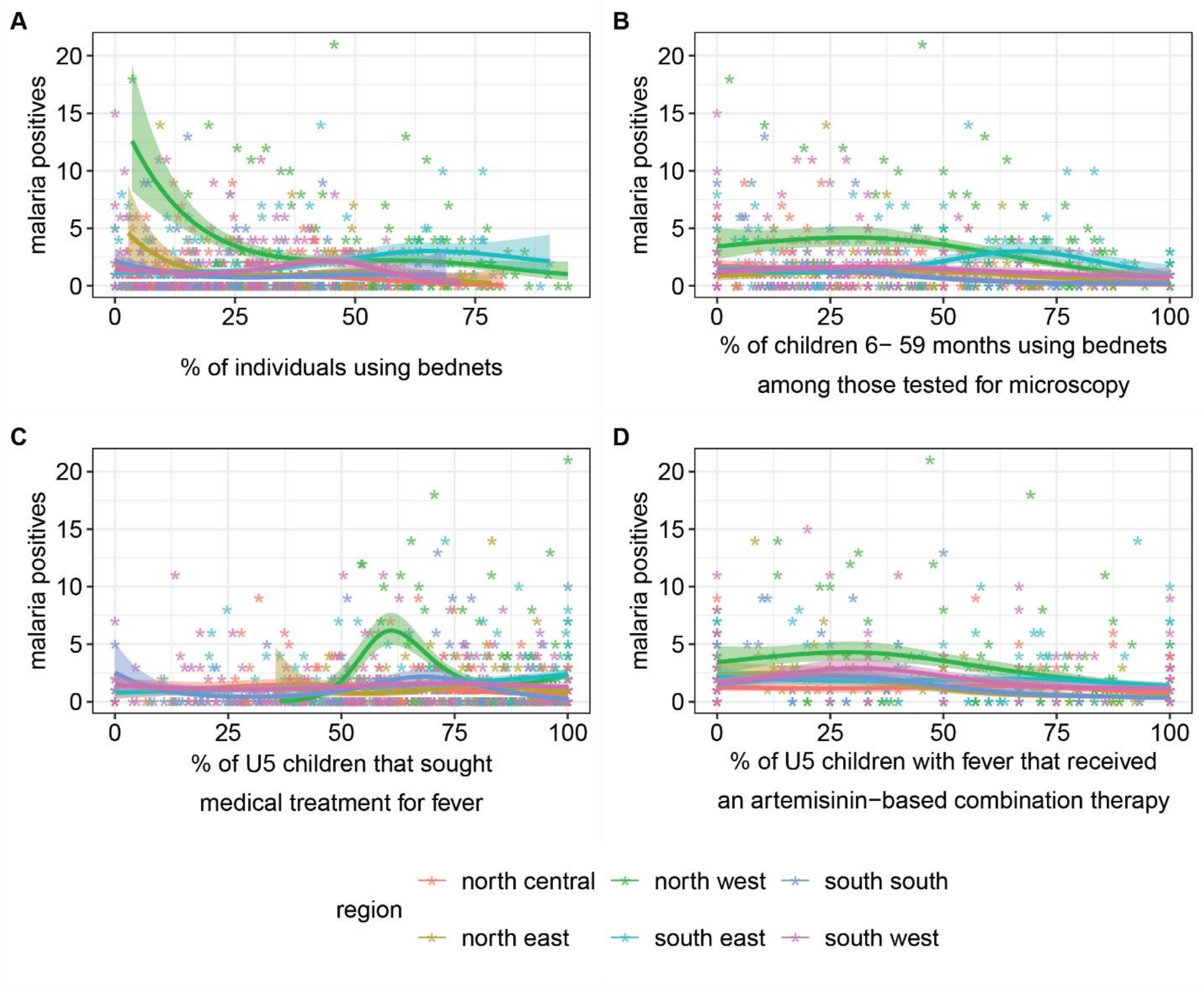

Supplementary Figure No: 19A – 19D. Associations between malaria positives and behavioral covariates, stratified by region. Stars, lines and shaded areas (95% confidence interval) are colored by geopolitical region. Fitted lines are based on a Poisson regression model fit. A) Percentage of individuals using bed nets (Bed net use is defined as those that slept under a net the night before the survey). B) Percentage of children using bed nets. C) Percentage of children under the age of five years that sought medical treatment for fever.

Accessibility factors

Descriptive analysis

Health care was highly accessible in most urban areas

The motorized travel time to health care in 2019 in 75% of clusters was 2.5 minutes (Supplementary Figure No: 20). Only 2 clusters had travel times within or exceeding one hour.

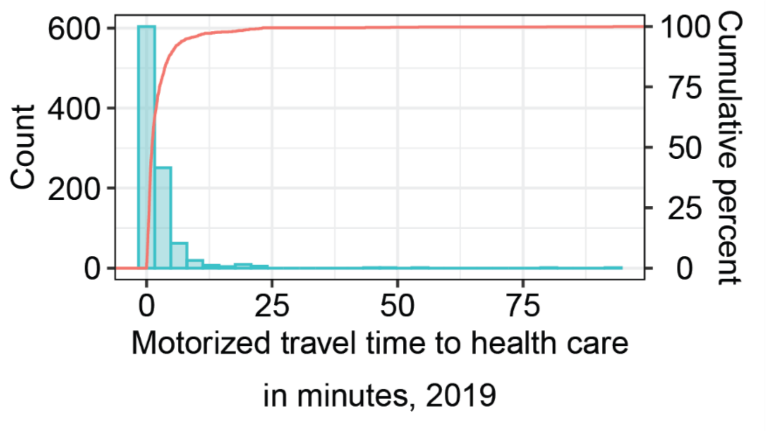

Supplementary Figure No: 20. Distribution of motorized travel time to health care for all cluster samples. The Supplementary Figure has two distribution illustrations and two y-axis. Turquoise histogram depicts the distribution of travel times. Left y-axis is the count values for the histogram. Red line is the cumulative distribution. Right y-axis is the cumulative percent for the red line.

Bivariate analysis

No impact of motorized travel time to health care on malaria transmission in urban settings

The fitted lines in the bivariate plots of the number of malaria positives against travel time was flat, which may indicate the absence of an association (Supplementary Figure No: 21a – c). However, in the Quasipoisson model, malaria test positivity rate increased negligibly between 0 and 5 minutes where most of the data was clustered (Supplementary Figure No: 21c).

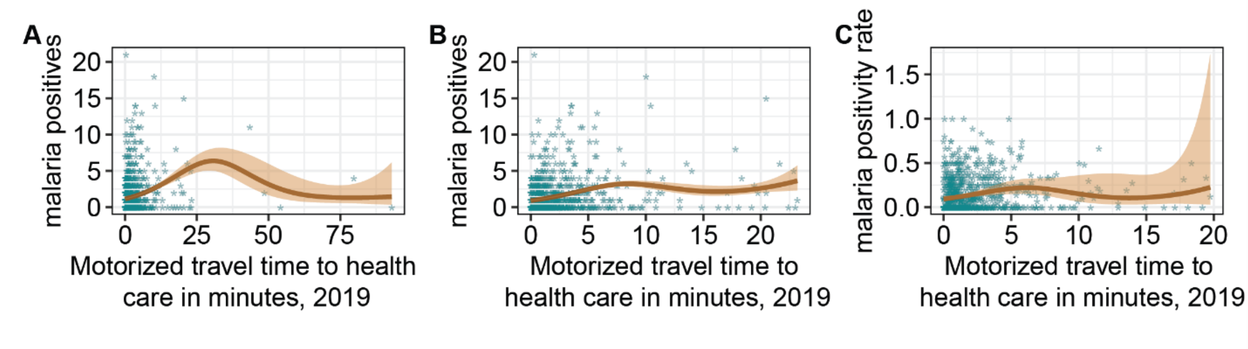

Supplementary Figure No: 21A – 21C. Bivariate associations with malaria positives and test positivity rate. A) Bivariate association of the travel times to health care with the number of malaria positives. Pale green stars are cluster data points. Brown lines and shaded areas are the Poisson regression model fit and 95% confidence intervals. B) Bivariate association of travel times to health care with the number of malaria positives. Due to right-skewedness, plotted travel times are limited to ≤ 25 minutes to more clearly visualize bivariate associations. C) Bivariate association of travel times to health care with malaria test positivity rate. Because the distribution is right skewed, values are limited to ≤ 20 minutes to enable visualization. Brown lines and shaded areas are the Quasipoisson regression model fit and 95% confidence intervals respectively.

Environmental factors

Descriptive analysis

Surveys months fell within the transmission season for the South-West, South-East and South-South geopolitical zones and temperature was mostly uniform across all clusters at the time of the survey

Total precipitation values estimated during the survey months of August to December were right-skewed and varied widely with 50% below 0.004 meters depth (IQR: 0.006 meters depth) (Supplementary Figure No: 22a). These values were lower than those of peak precipitation months such as July where median values were 0.007 meters depth in 2010, 0.006 in 2015, 0.006 in 2018, and 0.005 in 2021 (Supplementary Figure No: 23). Survey months, however, are within the malaria transmission

months for states in the South-West and South-East geopolitical zones where the start month of the malaria transmission season typically begins between March and April and ends in December [3]. States in South-South geopolitical zones experience year round transmission [3]. Temperature values showed little variation ranging from 25.4 ^{0}C to 27.2 ^{0}C in the first and third quartiles (Supplementary Figure No: 22b). Values of surface soil moisture per cluster, measured as gravimetric soil moisture, were left skewed with a median of 0.86 GSM (IQR: 0.25 GSM) (Supplementary Figure No: 8c). The maximum distance to water bodies was 10,000 meters, which is within 10,000 meters flight range of blood-fed *Anopheles gambiae* mosquitoes, the dominant malaria vector in Nigeria [4,5]. Most clusters had elevation estimates below 500 meters (Supplementary Figure No: 22e). The median enhanced vegetation index values was 0.3, and it ranged from 0.07 – 0.64.

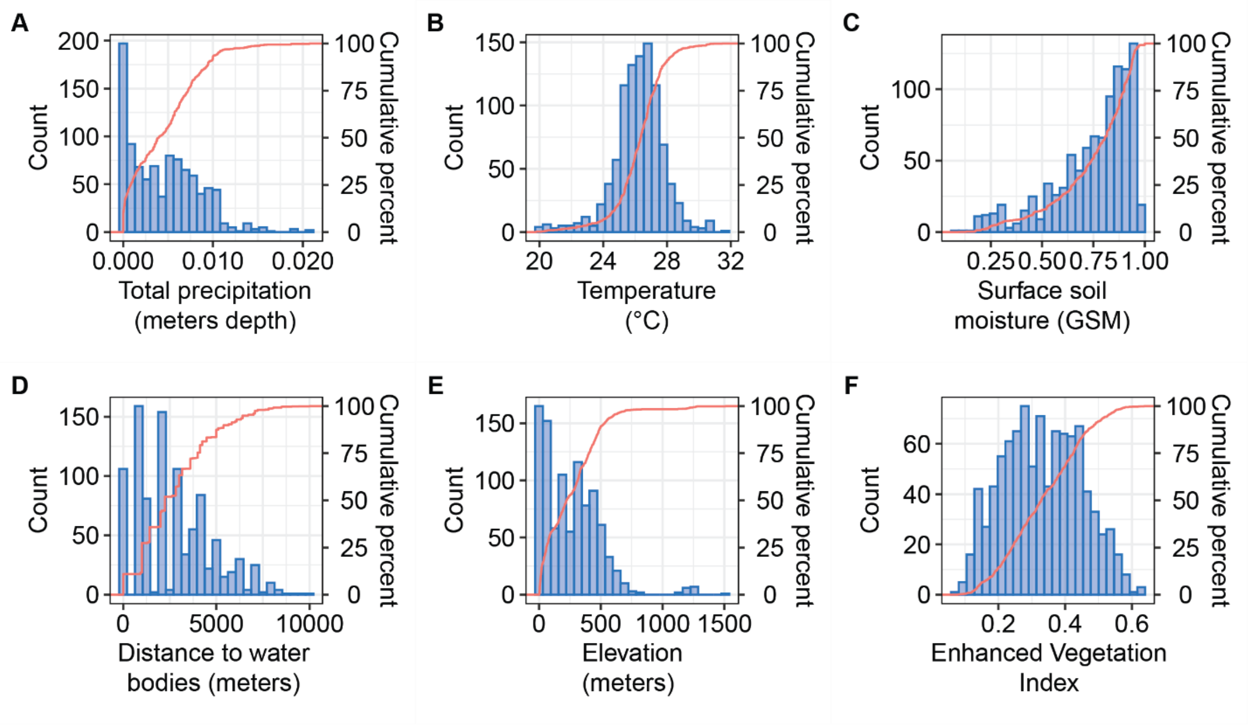

Supplementary Figure No: 22A – 22F. Distribution of environmental factors for all cluster samples. Each Supplementary Figure has two distribution illustrations and two y-axes. The blue histogram is each variable's frequency distribution while the red line is their cumulative distribution. The left y-axis corresponds to the number count for the blue histogram and the right y-axis corresponds to the cumulative percentage for the red line. A)

Total precipitation in units of meters depth. B) Temperature in degrees celsius. C) Surface soil moisture measured as gravimetric soil moisture (GSM) per cluster. GSM is the mass of water compared to the mass of solid materials per unit volume of soil. D) Distance to water bodies (meters). E) Elevation (meters). F) Enhanced Vegetation Index.

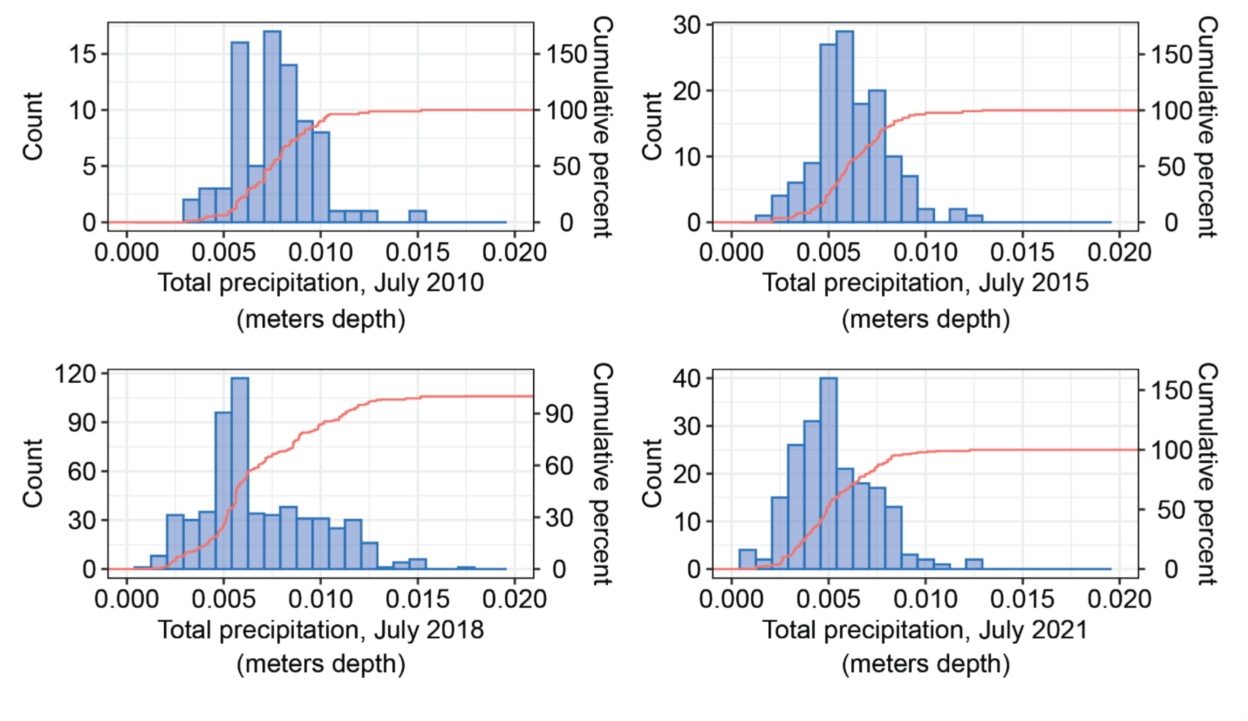

Supplementary Figure No: 23A – 23C: Distribution of total precipitation values in the month July for all 2010 – 2018 DHS clusters. Total precipitation values in July are typically higher than in other months. A) Total precipitation in July 2010. B) Total precipitation July 2015. C) Total precipitation July 2018.

Correlation matrix for environmental covariates

Temperature and elevation had a strongest negative correlation of 50%.

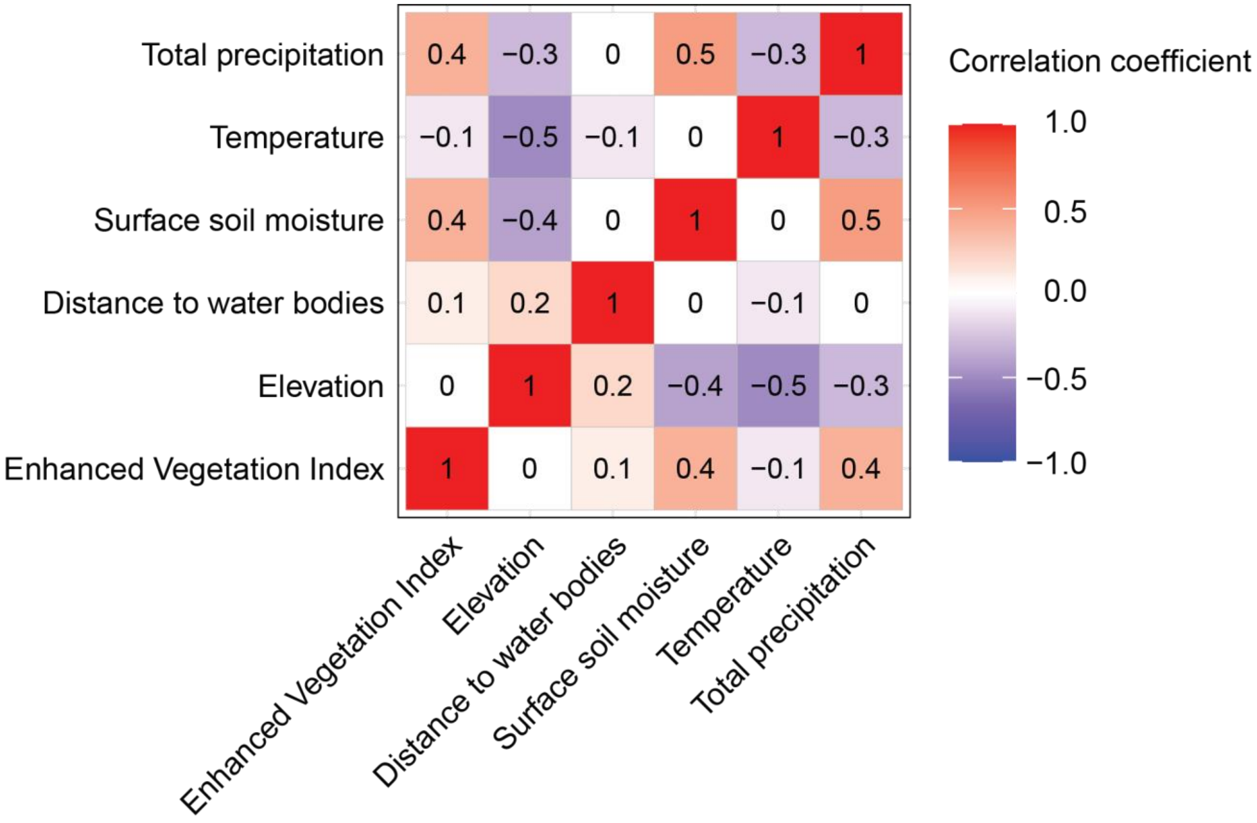

Supplementary Figure No: 24. Correlation matrix for environmental covariates. Numeric values in each box are Pearson's correlation coefficient. Temperature had a strongest negative correlation of 50% with elevation.

Bivariate analysis

Temperature maximally increases malaria transmission at around 30 ^{0}C while increases in the levels of enhanced vegetation index was positively correlated with small increases in transmission

The bivariate association between the number of malaria positives and most environmental variables were not particularly strong (Supplementary Figure No: 25). The fitted lines for precipitation, surface soil moisture, distance to water bodies and elevation, while not linear, were mostly flat. The predicted number of malaria positives increased with rising temperature values, dipping moderately around 24^{0} C

through 26^0 C before peaking at the highest temperature values of around 30^0. Increases in enhanced vegetation index (EVI) correlated with small increases in the number of malaria positives. Modeling the malaria test positivity rate paints a slightly different picture due to the normalized y-axis (Supplementary Figure No: 25). Temperature and EVI show a more obvious non-linear relationship with test positivity rate, albeit with higher levels of uncertainty.

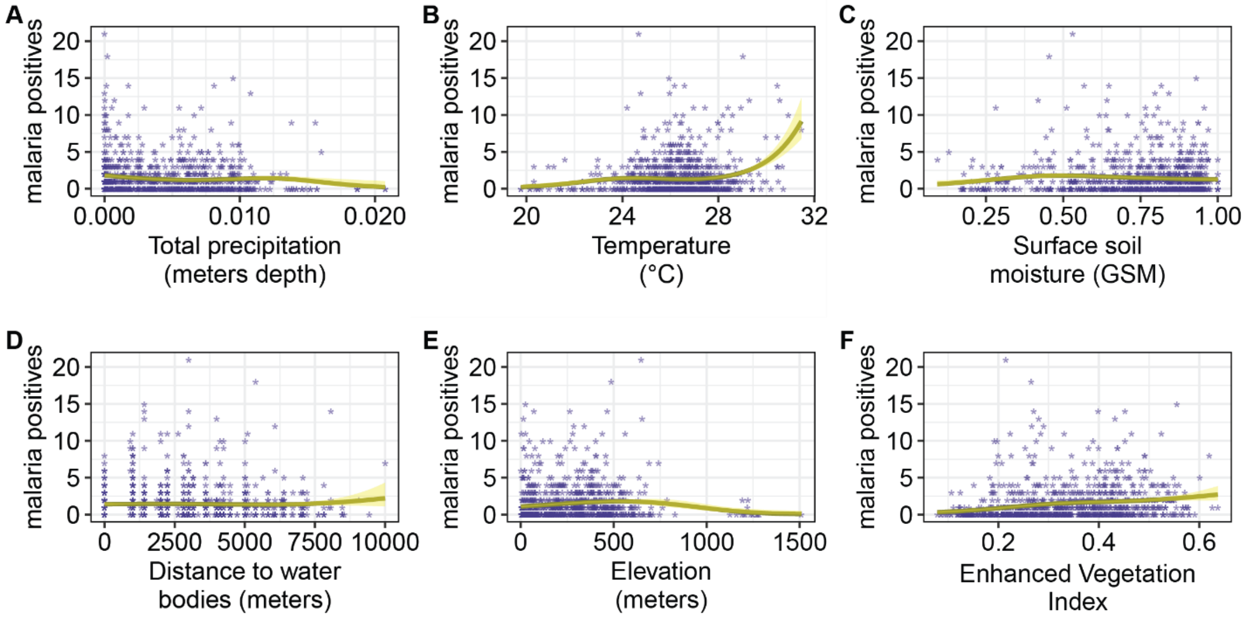

Supplementary Figure No: 25A – 25F. Bivariate association between the number of malaria positives per cluster and environmental covariates. Deep purple stars are cluster data points. Deep pink lines and shaded areas are the Poisson regression model fit and 95% confidence intervals respectively. A) Precipitation in units of meters depth. B) Temperature in degrees celsius. C) Surface soil moisture measured as gravimetric soil moisture (GSM) per cluster. GSM is the mass of water compared to the mass of solid materials per unit volume of soil. D) Distance to water bodies (meters). E) Elevation (meters). F) Enhanced Vegetation Index.

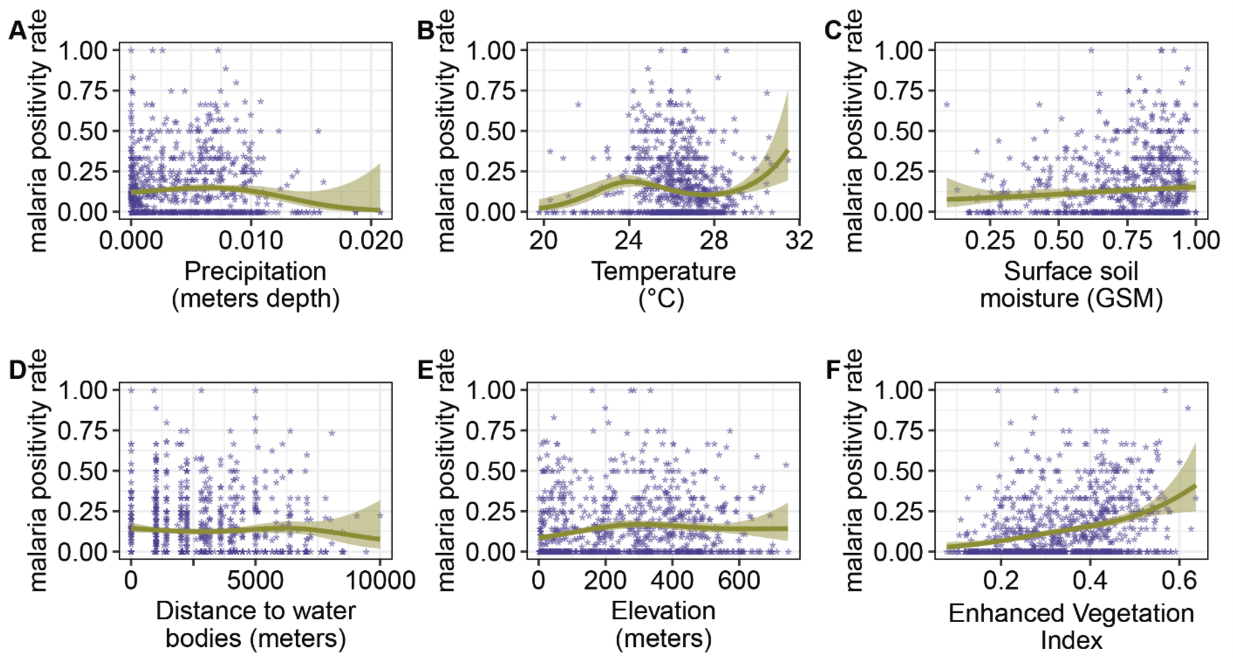

Supplementary Figure No: 26A – 26F. Bivariate association between the number of malaria positives per cluster and environmental covariates. Deep purple stars are cluster data points. Deep pink lines and shaded areas are the Quasipoisson regression model fit and 95% confidence intervals respectively. A) Total precipitation in units of meters depth. B) Temperature in degrees Celsius. C) Surface soil moisture measured as gravimetric soil moisture (GSM) per cluster. GSM is the mass of water compared to the mass of solid materials per unit volume of soil. D) Distance to water bodies (meters). E) Elevation (meters). F) Enhanced Vegetation Index.

Model diagnostics plots

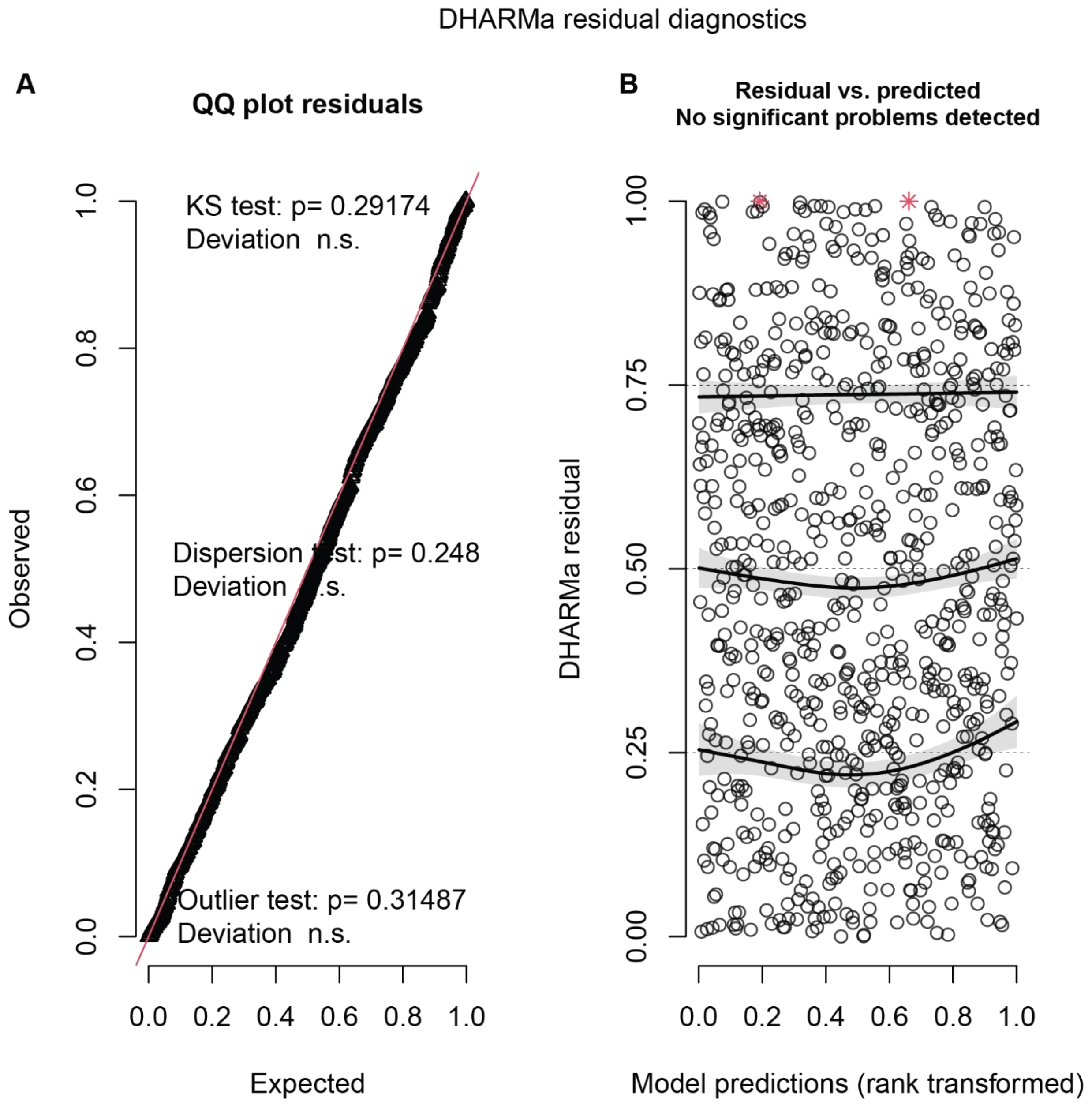

Supplementary Figure No: 27A – 27B. Model diagnostic plots. A) QQ plot of the observed and expected data distribution assuming the possion model fit is correctly specified. The KS test suggests that deviations from the empirical distribution are not significant. B) Plot of the residuals against the predicted values. No significant problems from the empirical quantiles were observed

Supplementary Table S1: Multivariable regression summary results for fixed effects

Term	Estimate	Standard error	*P*-value
(Intercept)	-1,72	0,49	0,00
North-East geopolitical region	-0,76	0,19	0,00
North-West geopolitical region	0,24	0,16	0,15
South-East geopolitical region	0,27	0,17	0,12
South-South geopolitical region	0,45	0,21	0,03
South-West geopolitical region	0,81	0,17	0,00
% with post-primary education (knot 1)	-0,53	0,31	0,09
% with post-primary education (knot 2)	-1,04	0,29	0,00
% with post-primary education (knot 3)	-1,20	0,49	0,01
% in the rich wealth quintiles (knot 1)	-0,35	0,20	0,08
% in the rich wealth quintiles (knot 2)	-1,98	0,38	0,00
% in the rich wealth quintiles (knot 3)	-0,83	0,18	0,00
% living in improved housing in 2015 (knot 1)	0,19	0,22	0,38
% living in improved housing in 2015 (knot 2)	0,28	0,45	0,53
% living in improved housing in 2015 (knot 3)	0,03	0,33	0,93
All age population density (knot 1)	-1,02	0,69	0,14
All age population density (knot 2)	0,79	0,91	0,39
Median age (knot 1)	-0,34	0,26	0,19
Median age (knot 2)	0,16	0,55	0,78
Median age (knot 3)	0,19	0,73	0,79
% of U5 children that sought medical treatment for fever (knot 1)	-0,42	0,17	0,01
% of U5 children that sought medical treatment for fever (knot 2)	-0,07	0,24	0,75
% of U5 children that sought medical treatment for fever (knot 3)	-0,30	0,12	0,01
Total precipitation (knot 1)	0,03	0,30	0,91
Total precipitation (knot 2)	-1,20	0,48	0,01
Total precipitation (knot 3)	-1,04	0,57	0,07
Enhanced vegetation index (knot 1)	0,92	0,32	0,00
Enhanced vegetation index (knot 2)	1,14	0,92	0,22
Enhanced vegetation index (knot 3)	0,66	0,42	0,12

References

1. National Population Commission (NPC) [Nigeria] and ICF. *Nigeria Demographic and Health Survey 2018*; Abuja, Nigeria; Rockville, MD, USA ; 2019. National Population Commission, Abuja, Nigeria.

2. National Malaria Elimination Programme. National Malaria Strategic Plan 2014 - 2020. 2013. Available online: https://extranet.who.int/countryplanningcycles/sites/default/files/ planning_cycle_repository/nigeria/nigeria_national_malaria_strategic_plan.pdf (accessed on 12 December 2020).

3. National Malaria Control Programme, suMAP, World Health Organization, INFORM Project. A description of the epidemiology of malaria to guide the planning of control in Nigeria. In A *Report Prepared for the Federal Ministry of Health, Nigeria, the Roll Back Malaria Partnership and the Department for International Development, UK*; 2013. National Malaria Control Programme, Abuja, Nigeria.

4. Akpan GE, Adepoju KA, Oladosu OR, Adelabu SA. Dominant malaria vector species in Nigeria: Modelling potential distribution of Anopheles gambiae sensu lato and its siblings with MaxEnt. *PLoS One*. 2018, https://doi.org/10.1371/journal.pone.0204233

5. Kaufmann C, Briegel H. Flight performance of the malaria vectors Anopheles gambiae and Anopheles atroparvus. *J Vector Ecol.* 2004;29: 140–53. https://pubmed.ncbi.nlm.nih.gov/15266751/
